# On the sustainability of an activity

**DOI:** 10.1038/srep05215

**Published:** 2014-06-12

**Authors:** Daniel S. Zachary

**Affiliations:** 1 Resources Centre for Environmental Technologies, Public Research Centre Henri Tudor, 29, avenue J.F. Kennedy, Grand Duchy of Luxembourg; 2 Current address: Whiting School of Engineering, The Johns Hopkins University, 6810 Deerpath Road, #100 Elkridge, MD 21076.

## Abstract

This paper develops a framework to determine the sustainability of a general activity. We define an activity as an action or process that uses one or more resources and that responds either wholly or partially to a demand. A definition for sustainability is developed and is contingent on whether or not an activity can be sustained according to the available resources, the duration of an activity, the cost of its execution, or whether substitution is possible. A sustainability condition is met when the duration, cost and the chain of dependent activities satisfies the demand. Two conditions for sustainability are developed: a strong condition when the demand is met with no substitution and a weak condition when the demand is met via substitution. In the latter case, we show that the set of all sustainable activities is a subset of a N-level union of sustainable activities and forms a topological cover.

In the 21st Century, humans are faced with the extraordinary challenge of developing methodologies to manage Earth's limited resources in a sustainable way^1^,^2^,^3^,^4^. A widely accepted definition of sustainability has its origins in the Brundtland Commission statement of 1987, *Our Common Future*^5^: “Development that meets the needs of the present without compromising the ability of future generations to meet their own needs.” This normative description summarizes the document and proposes a set of fundamental qualities for sustainability, including the notion of being global (encompassing everyone) and providing for intra- and intergenerational equity and justice. This document proposes the reduction of inequities for all as well as providing a way to protect the common good for the poorest and wealthiest nations, not only for the present, but also for future generations. Indeed the very notion of sustainability, ‘needs to be sustained’ in light of the self-evident prospect that we have a limited supply of available resources.

As a consequence, scientists continue to seek a better understanding of the fundamental character of the interactions between nature and society and to explore whether these interactions are along sustainable trajectories. Policy makers have also tried to implement these concepts. Kofi Annan's report to the United Nations, *We the peoples, The role of the United Nations in the 21st Century*^6^, echoes this notion. Activated by an international effort, Annan claimed, “globalization must be built on the great enabling force of the market … and requiring a broader effort to create a shared future, based upon our common humanity in all its diversity.” In the years following the Brundtland Report, a set of three pillars (we use interchangeably with the term dimensions) were conceived, namely, the environmental, economic, and social pillars. Then in 1992, the United Nations held the “The Earth Summit” conference in Rio de Janeiro. It was here that the agenda for the 21st century was established (Agenda 21) and the ‘triplet’ was solidified into the well known Venn diagram that has since become an icon for visualizing sustainability.

In the attempts to connect the potentially conflicting issues from each dimension, many subsequent interpretations and a host of definitions based on different academic disciplinary perspectives, ideological preferences, [and] even political expediency were developed. Some well known and constructive definitions include those for sustainable production^1^, sustainable biophysical systems^2^, eco-systems goods and services^4^, and others. Using some of these definitions, holistic approaches have been sought in response to the diverse challenges^7^ and have had some success in building frameworks to address sustainability problems.

There is a clear imbalance in terms of analytical work on the three dimensions. Environmental and the closely-related ecological sciences, most naturally accessible to modelling, have developed the first among the three^8^,^9^,^10^,^11^, followed by quantitative economic approaches towards sustainability^12^, and then much later by work on social sustainability^13^. Social sustainability is also commonly understood as a ‘weak pillar’, in part because of the difficulty in formulating issues into an analytic framework. Some progress has none-the-less been made^14^,^15^,^16^. Despite the differences of the three, there is a general agreement that holistic approaches to sustainable development must encompass 1) the interactions across all three dimensions, 2) multiscale approaches^17^, and 3) complexity^18^,^19^,^20^. Apart from these concerns, the modeller must balance detail and relevance since a model will only be as good as the available input information and the associated uncertainties. Forecasting sustainability trajectories is an even more difficult task when so many unknowns, both present and future, must be taken into account. A seemingly good solution for the present might turn out to be disastrous later on^3^. Currently, no clear methodology links the three dimensions, and no forecast method provides a vivid picture as to how we should use our limited resources.

In this paper, we develop a methodology linking the three dimensions and providing a means to distinguish between sustainable and non-sustainable activities. We provide a simple parametrization that connects renewable and non-renewable resources associated with the activities. In the next section, we recount the major historical steps in bridging sustainability dimensions.

## On the road to modelling sustainability

Early interdisciplinary work came in the 1940s, when Karl William Kapp, considered one of the founders of ecological economics (EE), proposed the connections between societal and economic activities^21^, “Social cost[s]… are all direct and indirect losses sustained by third persons or the general public as a result of unrestrained economic activities.” In these early years, the societal dimension was treated as a subsystem of the ecosystem. In the same time period, Karl Polanyi produced his work on *The Great Transformation* (1944)^22^ and developed the concept of societal and cultural economics. In the 1960s, a number of essays describing the interactions between the economic and ecosystems appeared, including Kenneth E. Boulding's work “The economics of the coming spaceship Earth”^23^ and Herman E. Daly's contribution entitled “On Economics as a life cycle”^24^. In the 1980s, a next major step came when regular scientific activities commenced in the field of EE. In 1984, the symposium entitled “Integrating Ecology and Economics” was hosted in Sweden by Ann-Mari Jansson^8^ and brought together, for the first time, a large number of ecosystem ecologists and mainstream environmental economists. In 1988, the International Society for Ecological Economics (ISEE) was founded, and, in February 1989, the first volume of Ecological Economics, the “Transdisciplinary Journal of the ISEE”, was published^26^. Today, EE is considered a well established scientific community. A good review of EE by Kastenhofer et al. (2011)^25^ provides an overview of this period and includes some of the more recent works^29^.

Also, during this period, the interdisciplinary fields of sustainability science (SS)^28^,^30^,^31^,^32^ emerged as a set of disciplines addressing the central issues of sustainability. A comprehensive definition is proposed by Kieffer et al. (2003)^33^: “[Sustainable science is] the cultivation, integration, and application of knowledge about Earth systems gained especially from the holistic and historical sciences ... coordinated with knowledge about human interrelationships gained from the social sciences and humanities, in order to evaluate, mitigate, and minimize the consequences”.

Despite the definitions available, no consensus on a theoretical framework has yet been defined in either interdisciplinary movements^25^,^27^. A current priority theme is the development of strategies dealing with the dynamical interfaces between dimensions^25^,^34^ and the uncertainties of measurable parameters.

A modern technique in dealing with the problem of interfaces has been the use of systems approaches (SA) where tools have traditionally been used to treat two or more domains of research. In SA, different metrics for each discipline are treated separately^3^. The method uses dynamic processes^35^ (time dependent), is cross dimensional (economic, social, and environmental) and is able to handle complex problems^36^ (interconnected, interwoven, with feedback) as well as problems with multi-variables. As with most approaches dealing with complex issues, SA employs numerical methods that can handle problems of this nature, but these approaches are not always easily interpretable.

Another quantitative method includes the use of sustainability assessment maps (SAMs). These maps use indicators such as costs, profits, numbers of jobs, environmental impact and types and quantities of natural capital. A score is set up to weigh each decision in terms of its sustainability^3^. The approach is appealing for policy makers because it is easy to communicate and understand, although it should not be considered a panacea^37^. Some problems include the aforementioned ‘imbalance between ecological modelling and the other two branches’^8^,^9^,^10^,^11^, the weak integration of data into a single, dynamical framework since SAMs are static^37^, the unresolved issue of what to do when conflicting goals and interactions between indicators have not been sufficiently considered, and finally, the mismatch of scales (local to global). The last issues is especially important in the social dimension^11^,^37^.

Several other approaches have been pursued to overcome these challenges, including the multivariate analysis^38^ and comprehensive indicator approaches^39^, both having a certain degree of success in specific applications^37^. The issues of complexity, non-locality, and multi-scales, still proved difficult to address with these methods, both in terms of calculation and interpretation. It is not surprising that no single approach for modelling sustainability has yet emerged. Furthermore, no consensus exists for a comprehensive list of sustainability topics. A good compilation of theoretical approaches has nonetheless been developed (adapted from van den Bergh, 1996)^40^ and is given in Table 1.

The previously mentioned approaches suggest the inter-connectivity of sustainability. An underlying concept connecting the dimensions is *cost*. This is not necessarily a monetary value, except in the obvious case of commodities in the economic dimension, but a parameter used to weight and compare decisions. Monetary values are more difficult to assign to environmental costs, although methodology has been developed to do so^41^,^42^. Impact has also been evaluated in other contexts, for example, direct human activity in the environment via population growth^43^. Also, not surprisingly, it is difficult to assign a monetary cost for societal concerns, but a relative weighting can still be done, especially for issues at a local level where a consensus on the social cost is easier to obtain.

For example, when dealing only with energy resources and long-term planning, the model should provide solutions that approach those of classic technoeconomic models (e.g., MARKAL, the MARket ALlocation model)^44^. When details are available (e.g., costs of fuel, maintenance, and investments), the model should be readily adaptable to search for and test different hypotheses. Indeed, the model should provide a means to make decisions or at least be flexible enough so that a decision process can be developed around the core model.

The model should also be adaptable to handle dynamic systems such as the dynamic resource stocks of limited resources (e.g., fossil fuels) or linked (indirect) resources typically modelled in ecological - economic literature (e.g., tuna: the harvesting of this top predator fish and its dependency on herrings).

Environmental costs, though less tangible, can also be determined via an impact or damage. A number of air pollutants, including CO_2_, nitrous oxides, hydrocarbons, and particulate matter are emitted from the vehicle and damage or impact the health of the surrounding human and plant environment. Although difficult to calculate on an individual basis, the damage to humans can be assessed in terms of the health costs or decreased longevity as a result of the diminished environment^42^.

The social costs depend on the social milieu. For example, the social acceptability of taking the bike instead of the car, may or may not be important. In some localities, residents consider it to be more socially responsible to use a bike for a short trip, especially if the other choice was an oversized car; yet in other societies, for cultural reasons, the consideration is just the opposite. This reality suggests that social costs may still be evaluated, but more as a means of weighing two or more decisions based on their local acceptance.

In this paper, we break down the concept of sustainable development into its building block activities; these can then be tested for the sustainability condition as we will show in the next few sections. Furthermore, we propose that sustainable development occurs only when the ensemble of activities that make up the development are all sustainable.

## Time scale considerations

Some activities will have immediate and direct consequences on their surroundings; others will have delayed impact. It is therefore important to model the activity with its associated impact and cost and capture both the immediate and the future consequences. The temporal behavior of activities and their costs must therefore be taken into account and modeled in a way that also reflects the limited knowledge that is available. We are now prepared to provide a general approach.

## Properties of an activity level

We define a set of *n* time-dependent *activities*, *i*, with label *a_i_*(*t*), *i* = 1, …, *N*, each with capacity *c_i_*(*t*), *c_i_*(*t*) = *∂x_i_*(*t*)/*∂t*, 
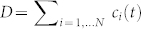
. We use the notion of capacity as that *required* or needed to meet a certain demand, contrary to the *available* capacity that may exceed the requirements of the demand. Consequently the resource *x_i_* is diminished according to this rate in response to meeting a single demand *D*.

An activity is equivalent to a set of actions or processes with a base *ℓ* = 1, and *ℓ* = 2, …, *N* representing substitution or replacement activities. To lighten the notation, we hereafter drop the time in the following definitions.

### Definition 1

*A general activity*, 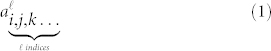
*is ℓ − 1 removed from the base activity and has capacity *
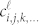
*, using resources *
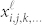
*. The substitution level ℓ has an equivalent number of running indices i, j, k, ….*

Base activities 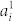, *i* = 1, … *n_i_* and substitutes *ℓ* = 2, 3, … are defined for each *i*. The first substitute activities are 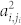 *j_i_ : = 1, …, n_i_*_,*j*_, etc. A level-dependent *n* replaces *N*. To lighten the notation, we drop the *i* subscript on *j* hereafter.

**Figure 1:**
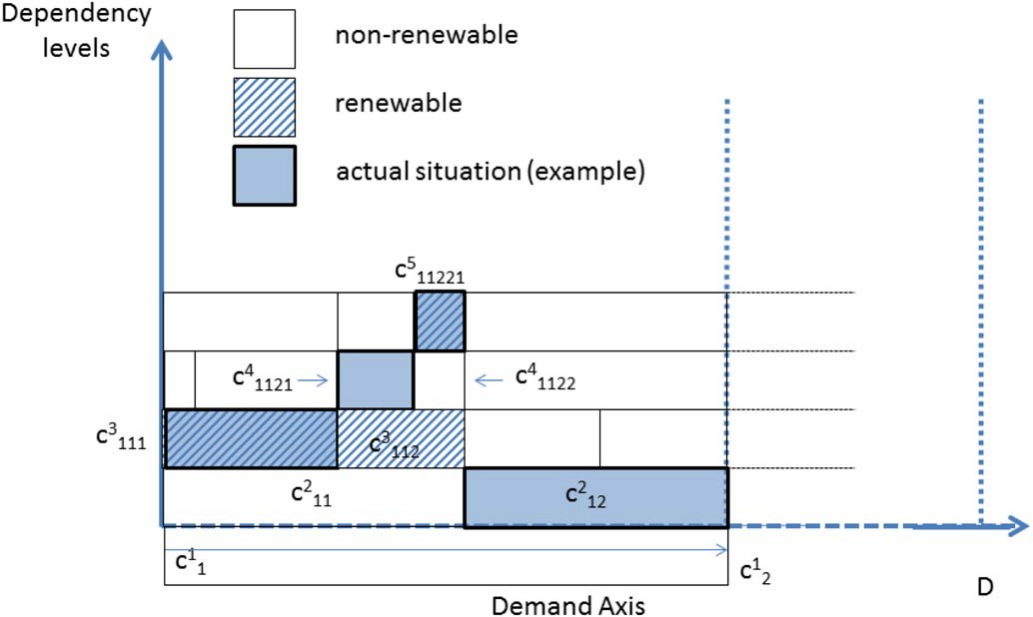
Schematic of dependencies (*N* = 5) for the base activity 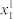 using capacity 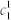.

Returning to our short trip example, supposing that the bike is chosen as the mode of transportation, then this single activity meets the demand, *c*_1_ [Joules] = *D* [Joules], fulfilled by the consumption of an apple. An activity may require several resources. For example, a multiple list of objects, representing the four basic food groups, 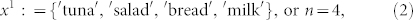
might be desirable to fulfill the needed demand. Substitution may occur here, if ‘tuna’ 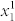 is not available but a another fish (e.g., salmon or grouper) is and would be designated 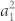. This example is time independent. An example of a base activity and three levels of dependencies are sketched in Figure 1. Here we show one capacity at the base level 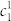 and its substitutes up through level five. Both renewable and non-renewable resources are shown along with a proposed actual example.

### Definition 2

*The activity *
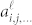
* occurs over a duration *
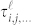
* and has a maximum allowable duration *
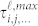
*,*

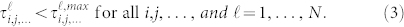
This condition ensures that each activity occurs in a feasible time period defined either when the activity is available or when resources are available.

Two assumptions have been made: First, we have considered a nonrenewable resource. A time dependent and renewable stock (e.g., a fish population), can be treated in this same framework, but with additional considerations on 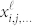, see Appendix (A dynamic reservoir). Second, we have assumed that only one activity is associated with a resource. In reality, several activities can be linked to a single resource and, in this case, we need to partition the resource for each activity. A multiple and simultaneous set of demands can be subsequently met by individual resources *x_i_*. Petroleum meeting the demand for transportation *x_petrol_*_→*trans*_ and petroleum used as raw material for the plastic industry, *x_petrol_*_→*plastic*_, is an example. The resource *x_petrol_*_→*trans*_ is implicitly used only for transportation while *x_petrol_*_→*plastic*_ is implicitly only used for plastics, *x_totpetrol_* = *x_petrol_*_→*trans*_ + *x_petrol_*_→*plastic*_. The uncertainty in the demand for transportation and plastics implies that some of the petroleum reservoir initially allocated for transportation could be used for the plastic industry and visa-versa. The time dependency on the activity, the cost, the demand, and any other parameters is implicit and generally not known. For these reasons, only a limited amount of modeling can be done for long-term environmental impact and cost.

The cost *C_i_* for activity *a_i_* for a one-level dependent activity (temporarily dropping the activity subscript) is composed of three parts: economic *C_F_*, social *C_S_*, and environmental *C_E_*. For extended planning, the technoeconomic costs include the standard investment, operational (maintenance), energy and replacement (salvage) values. The environmental cost *C_E_* is proportional to the impact *I*, the pollutant *p*, and ultimately the required capacity *c*, using a set of conversion constants, *α*, *β*, and *γ*, 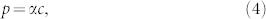

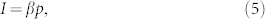

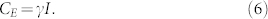
In this simple example where neither feedback nor other non-linear behavior occur, the environmental cost is directly proportional to the pollutant. Even in this example, the pollutant may accumulate over time and the net pollution *p*, resulting from the activity, can be diminished by dispersive action and/or by sequestration. Given a constant activity over a period of time *t*, the accumulated pollution *p_ACC_* and cost are, 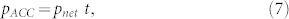

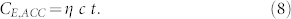
where *η* = *α* × *β* × *γ*. Behind this simple relationship lies environmental impacts and therefore costs having non-linear responses to the accumulated pollution levels resulting from complex physical processes and dynamic capacities.

Finally, social costs are even more challenging to model, but in principle, *C_S_*(*t*), if known or agreed upon, can also be included in a dynamic way.

### Definition 3

*The activity *
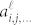
* using resources *
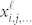
* has costs *
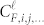
*, *
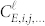
*, *
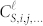
*, and maximum acceptable costs *
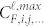
*, *
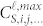
* and *
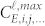
*,*

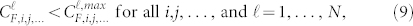

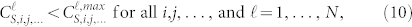

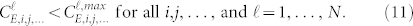


### Definition 4

*If either the duration, Eq. 2, or the costs, Eqs. 9 – 11, are not satisfied, then substitution may occur if it is available,*

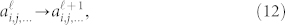

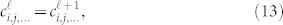
*iff, *
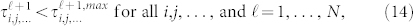
*and*

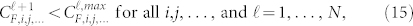

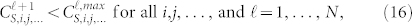

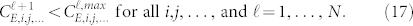
Though it is possible that 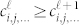, there is no guarantee that any additional capacity will be useful for other activities. For example, replacing one type of bread with another bread is acceptable, yet a salad replaced with twice as much bread would not meet the requirement (demand) for providing the four food groups.

## A single level set of activities

**Figure 2:**
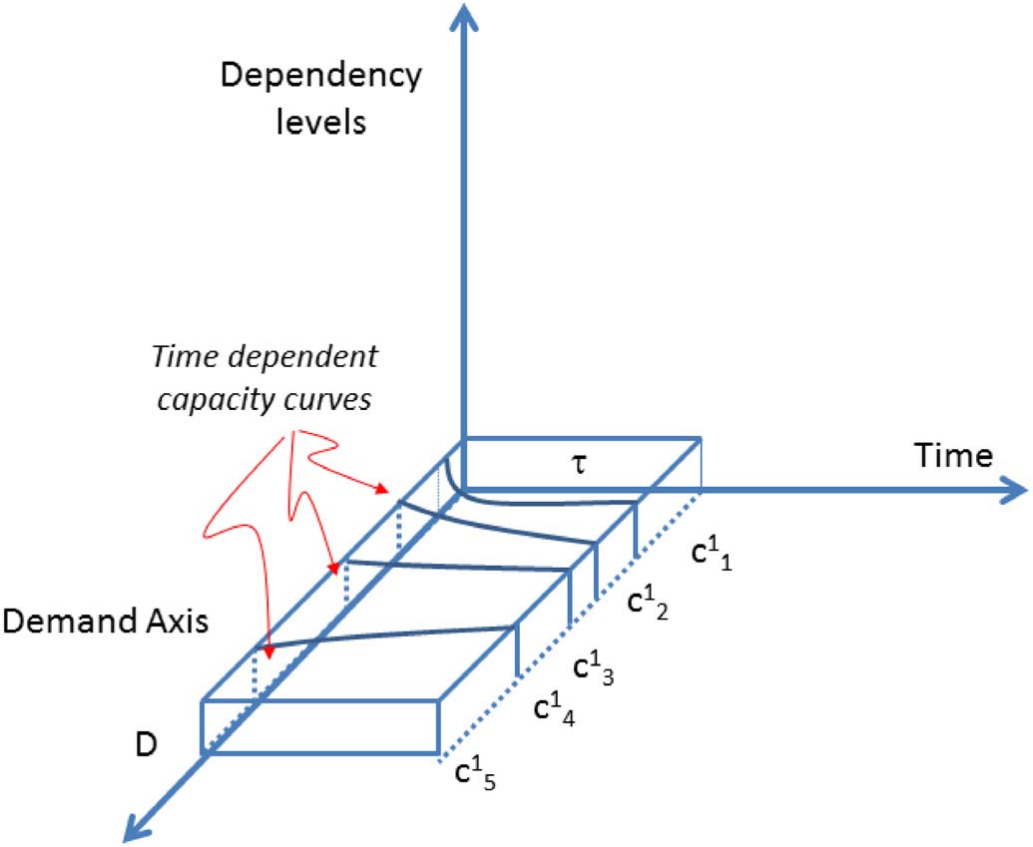
A set of single-level activities fulfilling a flat demand D.

Consider a set of *n* independent activities with only one level (no substitution possible), 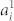, *i* = 1, … *n*, with capacities 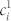, durations 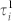, total cost 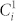, and each partially fulfilling a demand *D* in period *τ* = [*t_a_*, *t_b_*], then (using light notation), 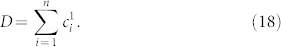
The time dependent activities of capacities 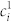 are sketched in Figure 2.

In this example, the capacities are time dependent but with equivalent duration *τ*.

## A multi-level set of activities

**Figure 3:**
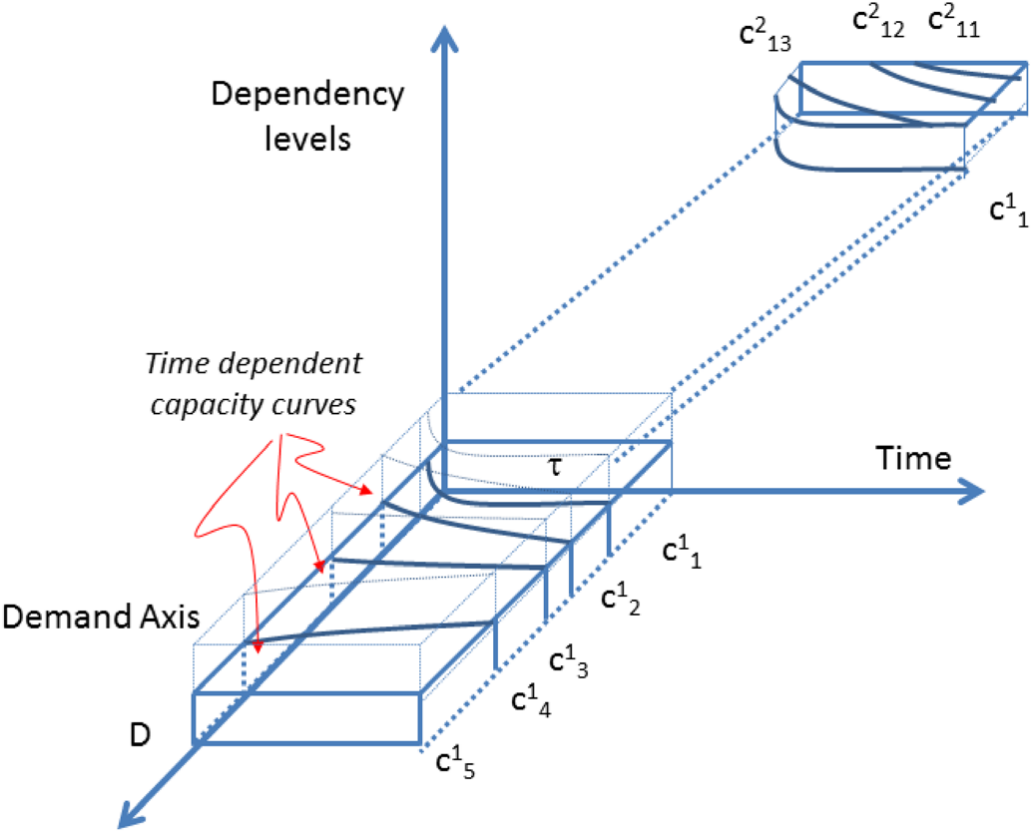
Multi-levels of activities and the dynamic connection between demand, activity, dependency, and time.

Consider a set of activities 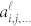 with *N* levels, each using resources, 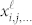 with capacities 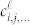 and with base activities 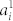 each partially fulfilling a demand *D* in a period *τ*. We now distinguish between the set of all sustainable 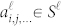, unsustainable 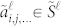, and total activities 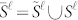. A demand *D* is fulfilled when there exists a combination of activities, via substitution, 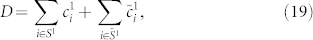

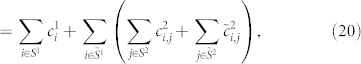

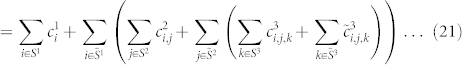

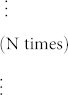

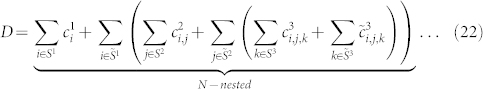
Equation 22 is truncated on the third term, and therefore, the unsustainable term shows up in this example and is assumed to be replaced by either sustainable or unsustainable capacities of higher levels, if they exit. Then the set capacities, represented by the activities 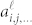 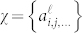, for all *i*, *j*, …, and all *ℓ* = 1, …, *N*, is a collection of (sustainable) sets, and if, 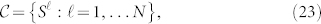
is an indexed family of sets *S^ℓ^*, then 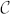 is a topological cover of *χ* if 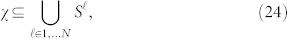
an *N*-level union of *S^ℓ^*. A similar argument can be made for the unsustainable set 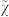. Figure 3 sketches a dynamic, multi-layer picture of activities and their dependencies. Here, details of a second level are also shown. Combining Definitions 1, 2, and 4 and Eqs. 22, we propose a condition for sustainability.

### Proposition 1

*Demand D, satisfied by sustainable activities *
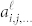
*, with capacities *
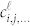
*, in period τ,*

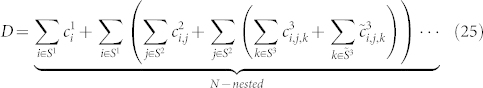
*is sustainable if the following conditions are met*, 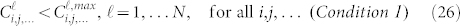

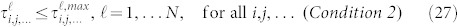
*and *
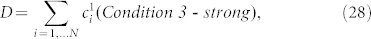
*or if the collection of sustainable sets,*

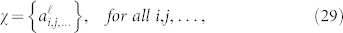

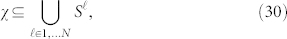
*where *
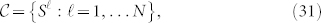
*where *
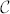
* is a cover of χ, and* *χ is a set of activities with capacities c, such that, *
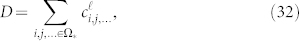
*where *
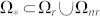
*, represents all i, j, … that fulfill the demand;* Ω*_r_ and* Ω*_nr_ are the sets of renewables and non-renewables available to* Ω_*_. Subsequently, χ is a subset of an N-level union of S^ℓ^. If Conditions 1 – 3 hold then D is fulfilled by sustainable activities, a, either directly (Condition 3 - strong sustainability) or by substitution (Condition 3 - weak sustainability).

These definitions are not to be confused with *Strong and Weak Sustainability* based on the use of natural capital^1^. A list of parameters used in the definitions is given in Table 2. Equations 22,23,24,26,26,27 do not represent an optimization problem, but by equating the activities to the demand, the formulation is structured similar to that of a dual solution typically found in cost minimization (linear program) problems with constraints.

## Examples of sustainable activities

Examples are useful to elaborate the notion of activities and their classification. We start with the simple example, the need (demand) to take a short trip and propose that only two modes of transportation are available: a bike and a car. First, we consider the resources required by each activity. Does the biker have enough stored bio-chemical energy available or is there enough gas in the car tank? If not, what are the costs of a snack for the biker to ‘energize up’ or the costs to refuel the car? If a longer trip was planned, we would have to include the purchase (replacement) and operational costs (maintenance) for both car and bike and include the amount recovered (salvage value) for each. For longer planning scenarios, covering decades of time, we must also consider the projected change of technology (e.g., a petroleum or diesel car replaced by a fuel cell car).

If the bike is chosen and the rider has enough energy to make the trip, then the trip is sustainable since *τ* < *τ^max^* and we assume *C_bike_* < *C^max^*. According to this scheme, the journey completed by a car is also sustainable if the car completes the journey before the tank is empty (*τ* < *τ^max^*) and *C_car_* < *C^max^*.

Other indirect consequences come out of this example. Theoretically, the ‘clean’ bike trip can negatively impact the environment. Prior to the trip, the bicyclist might be inclined to eat an apple for energy and therefore indirectly supports an apple farmer. A truck driver who is transporting the apple to the market is also supported by this activity, albeit marginally. The truck consequently contributes to the emission load. Therefore, secondary environmental costs that are not usually foreseen when riding a bike might be taken into account. To be thorough, we should not stop here; we should include the manufacturing costs needed to build the truck, bike, or car. We should also include the impact from the exhaust of all the commuters who journeyed to and from the factory to build the bike, car, or truck. The list would go on and on and the problem quickly becomes an intractable one to assess. Although arguably small, the ramifications of activities should be considered. To a degree, these are reminiscent of the Life Cycle Assessment considerations of the ‘cradle-to-grave’ analysis and Boundary Critique issues^45^ associated with typical rigid boundaries of problems. The model should be expandable to account for these issues by adding ‘higher-order terms’. The question of how these terms are weighted is probably less important than being consistent. (See Appendix item Generalizing the activity to include higher-order dependencies.)

One method to determine the higher-order terms in this case would be using the fraction of the weight of the one apple eaten compared to the weight of the entire lot on the truck. This information might be very difficult to ascertain and statistical models would be needed. The number of linked activities or levels *N* or the number of higher-order considerations will each have to be determined in light of the knowledge and uncertainty of the problem. When the higher-order term is sufficiently uncertain, the calculation should then stop.

Now consider two sets of capacities, renewables Ω*_r_* and non-renewables Ω*_nr_*, as given in Figure 1, 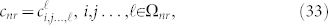

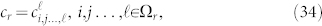

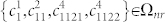
, 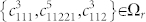, and as an example, an ‘actual’ situation, 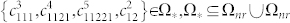. Using the capacities *c_r_* and *c_nr_* and current levels *c_r_*(0), *c_nr_*(0), 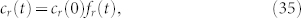

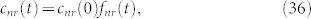
where *f_nr_*(*t*) and *f_r_*(*t*) are representative growth or decay functions for renewables and non-renewables and from Eq. 19, 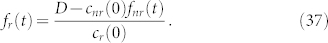
This example represents, among many others, a mixed resources problem with uniform evolution functions for renewable and non-renewable resources. Though the demand in this example is constant, it could be time dependent and therefore represents a national transportation activity. Scenarios developing replacement technologies (fuel cells) using renewable energies (solar, wind, etc.), for the most part, have not yet been developed on large scales, and therefore, the uncertainty of replaceable technologies limits the knowledge for substitution. Not surprisingly, this important demand is considered unsustainable and, subsequently, is a major area of concern and requires ongoing development and research.

Apart from the previously mentioned energy, transportation and ecological applications, other prime domains include agriculture, business, and architecture, and these can also be addressed with the model. A number of parameters and concepts can directly address the general definitions. Sustainable agriculture, for example, is characterized by a number of requirements and can be met by the framework developed here. These include:

1. Satisfying human food and fiber needs (activities meeting a demand, Eqs. 18, 19).
2. Enhancing environmental quality and the natural resource based upon the agricultural economy (reducing *I* and therefore *C_E_*, Eqs. 4–6).
3. Making the most efficient use of non-renewable resources and on-farm resources (replacing 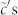 with higher level *c*'s or with other *c*'s having lower costs).
4. Integrating, where appropriate, natural biological cycles and controls (replacing 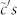 with higher level *c*'s or with other *c*'s having lower costs, Eqs. 19–22).
5. Sustaining the economic viability of farm operations (reducing *C_F_*, Eq. 9), and enhancing the quality of life for farmers and society as a whole (reducing *C_S_*, Eq. 10)^48^.

Sustainable business includes activities that are environmentally friendly processes, products, and manufacturing activities (reducing *C_E_*, Eqs. 4–6, 11) while maintaining a profit (reducing *C_F_*, Eq. 9)^49^.

Finally, sustainable architecture is typified by the promotion of smart growth and shorter commuting distances (reducing *C_E_* Eqs.4–6, 11), reducing the environmental footprint (or activities with acceptable *C_F_*, Eq. 10 and *C_E_*, Eqs. 4–6, 11), and avoidance of globally uniform design (reducing *C_S_*, Eq. 10).

## A classification of activities and relevance of the model

An activity is characterized by cost, duration, and whether it is a single or multi-level activity. A natural set of 2^3^ possibilities representing these combinations are given in Table 3. We note that the single or multi-level classification is determined by the activity using a resource (energy or stock) that is being depleted over the duration of the activity. This is distinguished from the wear or small depredation (e.g., tire wear during a bike ride) of the material in period *τ*.

A set of activities where higher-order terms can be important, such as the harvesting of fish (e.g., the Atlantic bluefin tuna), may be tested for sustainability according to the scheme. The harvesting of tuna will also affect the lower parts of the food chain (e.g., herring, mackerel, sardine), and in turn, these will affect even smaller fish. An unsustainable situation will arise if any of the links, all critical to the higher link, becomes unsustainable or if substitution was not possible at any of the endangered links. Unmanaged activity could lead to unfavourable consequences (e.g., overshoot and collapse of the tuna, mackerel, or sardine population).

## Discussion

Although the three-dimensional (pillar) view of sustainability is widely accepted, there remains a number of fundamental criticisms in terms of its modelling, especially in terms of the social pillar and its interfaces. Literature demonstrates a degree of criticism in terms of the interfaces, especially between the environmental and social dimensions^15^. Theys^46^ provides a way to proceed, noting that:

Local territories are the level at which the questions of socially sustainable development become concrete and where the interactions between the different dimensions are most explicit, and where participation and dialogue are the most feasible.

No single approach has yet emerged to bridge the gap between the local social issues, involving geographers, planners and landowners who deal with issues that immediately impact the local community, and global issues, involving economists, large enterprises, consumer organisations, international NGOs and diplomats who deal more with topics revolving around the global commons such as eco-taxes and emissions trading^46^,^15^. In the absence of well-defined analytic constructs such as the higher-order terms and social costs, social scientists will have difficulty connecting the local and global in an analytic way. We also note similar activities may have similar costs, and in this case a simplification could be made, e.g. *C_E_* = *C_E_*_,*i*_ for *i* representing two or more activities. Theys recommends a multi-tier system to address these separate social issues, similar to the indirect (higher-order) terms considered here.

Beyond this concern, connecting the social to the other dimensions is still problematic. An important study in the late 1990s was conducted by the Dutch Ministry of the Environment with the aim of gaining knowledge on the environmental and social interactions. A set of indicators was used to build an operational linkage between the social and environmental dimensions. As suspected, the study found that in some cases, social policies (e.g., reinforcement of consumption) were determined to adversely affect the environment (e.g., loss in eco-efficiency)^15^,^47^. The study concluded that in the absence of economic indicators, the model was too rudimentary to represent the complex interactions and causal relations between social and environmental indicators^15^. It was concluded that a holistic framework^47^ encompassing the three dimensions was still needed but that treatment of the social-environmental interaction was most easily handled, as previously mentioned, at the local level^46^. Translated to the approach presented here, the local or base activity *ℓ* = 1 is where social issues become more concrete. Perhaps consensus at the local level is one way to proceed. On the other hand, consensus of social costs on the global level, although more difficult, presents more noble ambitions in terms of tackling the issues of sustainability.

As noted earlier, a good model should be ‘falsifiable’ to the criteria it was meant to test; clearly some examples in the economic and environmental dimension are more readily testable than in the social dimension. Indeed, a proper balance must be achieved when providing numbers for costs, durations and substitutable activities, when knowledge permits. Only when these figures with their respective levels of uncertainty are tabulated can a full validation be determined.

Although numbers are not given here, Table 4 does provide a starting point in response to the not-so-quantifiable theoretical issues of sustainability. The terms *C_F_*, *C_E_*, *C_S_*, *C*, *τ_F_*, *τ_E_*, *τ_S_*, *τ*, the characterization of the activity *A*, and the possibility of substitution *S* indicates where the model could respond.

The model can be used to provide a response function for activities that use a mix of renewable and non-renewable resources. Any attempt to model these functions must consider the uncertainties in the dynamics of the demand and resources available, both for the present and the future.

Finally, we conclude that there is no panacea for solving all of the issues. Though the economic values can be determined from markets and environmental costs from measurements and models, the social costs will continue to be difficult to pin down. We observe from Table 1 the terms ‘balance’, ‘restrictions’, ‘maintaining’, ‘controlling’, ‘integrating’, ‘preventing’ are difficult to quantify. Some social issues, e.g., Socio-Biological, do not lend themselves easily to a quantitative model while others, e.g., Ecological Engineering, involving ecological resilience, do. Evidently, the role of uncertainty analysis will continue to play an important role in advancing the work and in connecting the dimensions in a coherent way.

## Summary and Conclusions

This paper adapts an analytic framework and approach in determining the sustainability of a general activity. Costs are used to connect the three dimensions and their theoretical maximum values, namely economic, social, and environmental. These maximums are used to test if the activity is sustainable. We also show that when cost and duration constraints are satisfied, an activity is classified as sustainable according to a strong condition (no substitution required) or weak condition (substitution required). We have shown that in the weak case, the set of all sustainable activities is a subset of an *N*-level union of sustainable activities, the set of which is a topological cover of sustainable activities. The number of levels is limited by the knowledge and uncertainty of the substitutable activities. A simple classification of activities is given, though quantification will be required to advance the work. What is surprising is that the basic framework is simple, closely following the three dimensions (pillars) of sustainability. While these three have been discussed for some time, the literature to connect them has been sparse. Perhaps this methodology will assist in their connection and allow for more quantitative studies in the field of sustainability science.

## Supplementary Material

Supplementary Information Supplementary Information

## Figures and Tables

**Table 1:** Adapted from van den Bergh^40^ ‘Theoretical Perspectives on Sustainable Development’

Theory	Characterization
1. Equilibrium-Neoclassical	Anthropocentric: welfare should be non-decreasing; SD should be based on technology and substitution; optimizing environmental externalities; the maintaining of aggregate stock of natural and economic capital; policy needed when individual objectives conflict.
2. Neo-Austrian-Temporal	Teleological sequence of conscious and goal-oriented adaptation; preventing irreversible patterns; maintaining organization level (negentropy) in economic system; optimizing dynamic processes of extraction, production, consumption, recycling and waste treatment.
3. Ecological-Evolutionary	Maintaining resilience of natural systems, allowing for fluctuation and cycles (regular destruction); learning from uncertainty in natural processes; no domination of food chains by humans; fostering balanced nutrient flows in ecosystems.
4. Evolutionary-Technological	Maintaining co-evolutionary adaptive capacity in terms of knowledge and technology to react to uncertainties; fostering economic diversity of actors, sectors and technologies.
5. Physico-Economic	Restrictions on materials and energy flows in/out of the economy; industrial metabolism based on materials product chain policy: integrated waste treatment, abatement, recycling and product development.
6. Biophysical-Energy	A steady state with minimum materials and energy throughput; maintaining physical and biological stocks and biodiversity; transition to energy systems with minimum pollution effects.
7. Systems-Ecological	Controlling direct and indirect human effects on ecosystems; balance between material inputs and outputs to human systems; minimum stress factors on ecosystems, both local and global.
8. Ecological Engineering	Integration of human benefits and environmental quality and functions by manipulation of ecosystems, utilizing resilience, self-organization, self-regulation and functions of natural systems for human purposes.
9. Human Ecology	Remain within the carrying capacity (logistic growth); limited scale of economy and population, consumption oriented toward basic needs; occupy a modest place within the ecosystem food web and biosphere; always consider multiplier effects of human actions in space and time.
10. Socio-Biological	Maintain cultural and social system of interactions with ecosystems; respect for nature integrated in culture; survival of group important.
11. Historical-Institutional	Equal attention to interests of nature, sectors and future generations; integrating institutional arrangements for economic and environmental policy; creating institutional long-run support for natures interests; holistic instead of partial solutions, based on a hierarchy of values.
12. Ethical-Utopian	New individual value systems and respect for nature and future generations, basic needs fulfilment, long-run policy based on changing values and encouraging citizen (altruistic) as opposed to individual (egoistic) behaviour.

**Table 2:** List of parameters and indices used in the model

Parameter	Definition
i,j,…	Indices for direct activities in level one (*i*), level two (*j*), etc.
*α*, *β*,…	Indices for first level indirect activities, second level indirect activities, etc.
*ℓ*	Level (of substitution).
a	Activity.
c	Capacity of activity.
x	Resource stock of activity.
*τ*	Duration of activity.
C*_F_*,C*_E_*,C*_S_*,	Cost of activity: economic, environmental, social.
D	Demand.
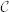	Cover of sustainable activities.
*S^ℓ^*, 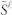 , 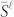	Set of all sustainable, unsustainable, and total activities of level *ℓ*.
*χ*, 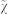 , 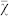	Set of all sustainable, unsustainable, and total activities.

**Table 3:** Eight possibilities for sustainable (S) or unsustainable (US) activities

Name	Level(s)	Duration	Cost
		*τ* < *τ^max^*	*C* < *C^max^*
US	single	no	no
US	single	no	yes
US	single	yes	no
S/US	single	yes	yes
US	multiple	no	no
US	multiple	no	yes
US	multiple	yes	no
S/US	multiple	yes	yes

**Table 4:** Response of the model to the theoretical perspectives (See text and Table 2 for symbols)

Theory	Model
1. Equilibrium-Neoclassical	*S*, *C_F_*, *C_E_*, *τ_F_*, *τ_E_*
2. Neo-Austrian-Temporal	*S*, *C_F_*, *C_E_*, *τ_F_*, *τ_E_*
3. Ecological-Evolutionary	*A*, *S*, *C_E_*, *τ_E_*
4. Evolutionary-Technological	*A*, *S*
5. Physico-Economic	*C_F_*, *C_E_*, *τ_F_*, *τ_E_*
6. Biophysical-Energy	*A*, *C_E_*, *τ_E_*
7. Systems-Ecological	*A*, *S*, *C_E_*, *τ_E_*
8. Ecological Engineering	*A*, *S*, *C_E_*, *C_S_*, *τ_E_*, *τ_S_*
9. Human Ecology	*A*, *S*, *C_E_*, *C_S_*, *τ_E_*, *τ_S_*
10. Socio-Biological	*C_E_*, *C_S_*, *τ_E_*, *τ_S_*
11. Historical-Institutional	*A*, *S*, *C*, *τ*
12. Ethical-Utopian	*A*, *S*, *C_E_*, *C_S_*, *τ_E_*, *τ_S_*
