# Supplementary material for: On the sustainability of an activity
Source: Sci Rep. 2014 Jun 12;4:5215. doi: 10.1038/srep05215 (PMC4054469; doi:10.1038/srep05215)
Supplement: Supplementary Information [file srep05215-s1.pdf]

On the sustainability of an activity (Supp)

Daniel S. Zachary

Appendix

A dynamic reservoir

A general model must be adaptable in terms of a dynamic reservoir, typical of fish populations in the context of harvesting (ecological-environmental models) [1]. For compactness of notation, we write the single level activity $x = x_i^1$ as the resource (stock) for an activity. If x depends on non-renewable resources, in the limit as $t \to \infty$, then

$$\lim_{t\to\infty} x(t) \to 0. \tag{A-1}$$

Likewise,

$$\lim_{t\to\infty} lgs(t) \to 0. \tag{A-2}$$

Then for every $\epsilon > 0$ there is a t^*, such that $t > t^*$, then $\epsilon > |x - 0|$. In other words, $x(t)$ can be as close to 0 as we desire, by making t sufficiently large. A similar argument can be made with $lgs(t)$. As both functions approach 0, $lim_{t\to\infty}|x(t) - lgs(t)| \to 0$.

However, it cannot be shown that resource x can only be described by the logistics function (managed activity) since a similar argument can be made for an unmanaged resource (e.g. the overshoot and collapse behaviour).

Generalizing the activity to include higher order dependencies

We define an indirect activity as one that depletes a resource $\lambda^{\ell}_{i,j,\ldots;\alpha,\beta,\ldots,}$, has capacity $\vartheta^{\ell}_{i,j,\ldots;\alpha,\beta,\ldots,}$, has a cost and duration, has a primary activity that is dependent on another, $\alpha = 1, \ldots n_{i;\alpha}, \beta = 1, \ldots n_{i;\alpha,\beta}$, and is similar in all other respects with Definitions 1 - 4. The additional subscripts are required for the indirect resources that are linked to a (direct) activity. As previously done, we lighten the notation, by suppressing the time dependence t. The demand of a single level, Eq. 14, is now refined,

$$D = \sum_{i=1}^{n} \left(c_i^1 + \sum_{\alpha=1,\ldots n_{i;\alpha}} \vartheta^1_{i;\alpha} \right), \tag{A-3}$$

where $n_{i;\alpha}$ is the number indirect resources associated with x_i^1,

$$\vartheta^1_{i;\alpha} = w^1_{i;\alpha} \cdot x_i^1, \tag{A-4}$$

and a weight, equivalent to the fraction of resource used by the primary activity x_i^1 and the total resource available at the higher order, $\lambda_{i;\alpha}^1$,

$$w_{i;\alpha}^1 \quad = \quad x_i^1/\lambda_{i;\alpha}^1. \tag{A-5}$$

In the truck and apple example, $w_{i;\alpha}^1$ represents the energy available in one apple x_i^1 compared to the energy available in the total number of apples carried on the truck, $\lambda_{i;\alpha}^1$. The definition can then be extended to an arbitrary activity level N,

$$D = \underbrace{\sum_{i\in S^1} c_i^1 + \sum_{i\in \tilde{S}^1}\left(\sum_{j\in S^2} c_{i,j}^2 + \sum_{j\in \tilde{S}^2}\left(\sum_{k\in S^3} c_{i,j,k}^3 + \sum_{k\in \tilde{S}^3} \tilde{c}_{i,j,k}^3\right)\right)\ldots}_{N-nested}$$
$$+ \underbrace{\sum_{i\in S^1} \vartheta_i^1 + \sum_{i\in \tilde{S}^1}\left(\sum_{j\in S^2} \vartheta_{i,j}^2 + \sum_{j\in \tilde{S}^2}\left(\sum_{k\in S^3} \vartheta_{i,j,k}^3 + \sum_{k\in \tilde{S}^3} \tilde{\vartheta}_{i,j,k}^3\right)\right)\ldots}_{N-nested}, \tag{A-6}$$

and beyond to third, fourth, ... levels, limited either by the lack of knowledge or the uncertainty in either the direct or indirect resources.

References

[1] Derissen, S., Quaas, M.F. & Baumärtner, S. The relationship between resilience and sustainability of ecological-economic systems, *Ecol. Econ.* **70**, 1121–1128 (2011).
